# Comparison of Low-Dose Rosuvastatin with Atorvastatin in Lipid-Lowering Efficacy and Safety in a High-Risk Pakistani Cohort: An Open-Label Randomized Trial

**DOI:** 10.1155/2014/875907

**Published:** 2014-03-30

**Authors:** Abdul Rehman Arshad

**Affiliations:** Department of Medicine, 1 Mountain Medical Battalion, Bagh, Azad Kashmir 12500, Pakistan

## Abstract

*Background*. Treatment of hyperlipidemia is helpful in both primary and secondary prevention of coronary heart disease and stroke. *Aim*. To compare lipid-lowering efficacy of rosuvastatin with atorvastatin. *Methodology*. This open-label randomized controlled trial was carried out at 1 Mountain Medical Battalion from September 2012 to August 2013 on patients with type 2 diabetes, hypertension, myocardial infarction, or stroke, meriting treatment with a statin. Those with secondary causes of dyslipidemia were excluded. Blood samples for estimation of serum total cholesterol, triglycerides, HDL-C, and LDL-C were collected after a 12-hour fast. Patients were randomly allocated to receive either atorvastatin 10 mg HS or rosuvastatin 5 mg HS daily. Lipid levels were rechecked after six weeks. *Results*. Atorvastatin was used in 63 patients and rosuvastatin in 66. There was a greater absolute and percent reduction in serum LDL-C levels with rosuvastatin as compared to atorvastatin (0.96 versus 0.54 mg/dL; *P* = 0.011 and 24.34 versus 13.66%; *P* = 0.045), whereas reduction in all other fractions was equal. Myalgias were seen in 5 (7.94%) patients treated with atorvastatin and 8 (12.12%) patients treated with rosuvastatin (*P*: 0.432). *Conclusion*. Rosuvastatin produces a greater reduction in serum LDL-C levels and should therefore be preferred over atorvastatin.

## 1. Introduction

Dyslipidemia is a well-recognized risk factor for the development of diseases associated with atherosclerosis, including coronary heart disease (CHD) and stroke. It has been estimated that almost every other adult in the United States has abnormal cholesterol values and every third person has elevated low-density lipoprotein cholesterol (LDL-C) levels [1]. Not only is the treatment of hyperlipidemia associated with improved outcomes in patients with these diseases, but also the lipid lowering is the most powerful intervention in primary prevention [2]. Statins are the first-line therapy for treating high lipid levels. In addition to the numeric reduction in lipid levels, they significantly reduce vascular events and all-cause mortality through their pleiotropic effects. It has already been proved that statins have antioxidant, anti-inflammatory effects and antithrombotic properties that add to their clinical utility [3]. They improve endothelial dysfunction and reduce the growth of atherosclerotic plaque [4]. Available evidence does not strongly suggest clear clinical benefit of other lipid-lowering agents in such situations [5].

All of the available statins have small differences in terms of pharmacokinetics and pharmacodynamics and hence in clinical efficacy and side effects profile [6]. Simvastatin and atorvastatin are the most commonly used ones [7]. Evidence from the Western countries suggests that rosuvastatin achieves greater reductions in LDL-C and has a higher rate of achieving therapeutic milestones than other statins [8]. However, such data from our country is limited and it is well known that Asians may respond differently from whites because of genetic differences in drug metabolism at the hepatic enzyme and drug transporter level [9]. This study was therefore carried out to determine the efficacy of rosuvastatin against the time-tested atorvastatin in a Pakistani cohort.

## 2. Patients and Methods

This randomized controlled trial was carried out at Department of Medicine, 1 Mountain Medical Battalion, Bagh, Azad Kashmir, Pakistan, from September 2012 to August 2013. Enrollment of patients started after an approval was obtained from the Ethics Review Committee of the Institute. Eligibility criteria included

- diabetics with myocardial infarction (MI) or stroke, regardless of baseline lipid levels;
- diabetics aged >40 years without atherosclerotic cardiovascular disease, but having an additional CHD risk factor (hypertension, smoking, obesity, male gender, and family history of premature CHD), regardless of baseline lipid levels;
- nondiabetic hypertensive patients were included in the study if lipid-lowering agents were indicated in accordance with the risk categories defined in National Cholesterol Education Plan-Adult Treatment Panel III (NCEP ATP III) guidelines:
  - hypertensive patients with MI or stroke;
  - hypertensive patients with LDL-C > 4.2 and smoking/high density lipoproteins (HDL-C) < 1.04/age > 45 years in men or >55 in women.

For patients already on any lipid-lowering agent, a six-week washout period had to pass before they could be considered for inclusion in this trial. Since stopping statins was not possible for some high-risk patients like those with acute myocardial infarction or stroke, they were obviously excluded from this trial. Other exclusion criteria included unwillingness of the patient and presence of any underlying condition producing dyslipidemia including hypothyroidism, nephrotic syndrome, biliary obstruction, and renal failure.

At the initial consultation, an informed written consent was obtained from the patients in their native language and demographic data was recorded. Blood samples were collected for estimation of serum total cholesterol, TG, and HDL-C after a 12-hour fast using Merck Microlab-300 Automated Clinical Chemistry Analyser. LDL-C was calculated using the Friedewald equation. Patients were then assigned to either of the two groups using nonprobability convenience sampling technique. The first group was to receive atorvastatin 10 mg before sleep (at bedtime) whereas the second group was to be given rosuvastatin 5 mg before sleep (at bedtime) every night, in addition to other medicines required for the underlying diseases. Statins were continued for a total of 6 weeks, during which period the patients were followed up on fortnightly basis. On each visit, compliance to treatment was assessed and possible side effects to treatment were recorded. Serum total cholesterol, TG, HDL-C, and LDL-C after a 12-hour fast were rechecked at the end of 6 weeks. In patients complaining of body aches and pains, serum creatine was checked to rule out myositis.

IBM SPSS Statistics Version 20 was used for intention-to-treat data analysis. All quantitative variables were described as mean ± standard deviation. Mean reductions in different lipid fractions in the two treatment groups over the six-week study period were calculated and then compared using independent samples *t*-test. Frequencies of patients developing different side effects were also calculated and compared between the two groups using chi-square test. A *P* value of ≤0.05 was considered significant for both of these tests.

## 3. Results

Out of 129 patients treated with statins during the study, 63 received atorvastatin, whereas 66 were given rosuvastatin. Their baseline characteristics are shown in Table 1 and the underlying diagnosis is shown in Table 2. Patients in the two groups had similar lipid levels to start with and, as depicted in Table 3, serum total cholesterol, TG, and HDL-C were reduced to the same level in both of the treatment groups. However, a significantly greater absolute and percentage reduction in serum LDL-C levels was observed in patients treated with rosuvastatin as compared to those treated with atorvastatin. Myalgias were the only side effect observed, seen in 5 (7.94%) patients treated with atorvastatin and 8 (12.12%) patients treated with rosuvastatin (*P*: 0.432). Since myalgias were tolerable and serum creatine kinase was normal in all of these patients (144.77 ± 21.83 U/L), statins were continued throughout the study period, albeit with close monitoring. None of the enrolled patients withdrew from the study due to an adverse event or by default.

## 4. Discussion

American College of Cardiology and American Heart Association have very recently issued guidelines on the treatment of blood cholesterol to reduce atherosclerotic cardiovascular risk in adults [10]. Prior to this, during the time period that this study was being carried out, clinicians were following NCEP-ATP III guidelines for lipid management [11]. During that era, LDL-C was the most significant lipid parameter and well-defined LDL-C and non-HDL goals for statin therapy existed depending on a 10-year CHD risk for a specific patient. This was in keeping with the fact that LDL-C is a major risk factor for coronary events, having a positive correlation even for levels in the normal range [12].

This study on Asian patients demonstrated an approximately 10% greater reduction in LDL-C levels with rosuvastatin. This superiority of rosuvastatin is in keeping with the findings of several other trials done on other racial groups. Notable amongst these is the landmark statin therapies for elevated lipid levels compared across doses to rosuvastatin (STELLAR) trial done on 2431 patients comparing rosuvastatin with atorvastatin, simvastatin, and pravastatin [13]. Across a wide dose range, rosuvastatin produced a significantly greater reduction in LDL-C levels as compared to its competitors. Similarly, Milionis et al. demonstrated a greater LDL-C lowering effect of 10 mg rosuvastatin as compared to that of 20 mg atorvastatin in patients with primary hyperlipidaemia [14]. Physicians should remain aware of the doses of different statins while applying the results of this present study to clinical practice. This is because different statins, with dose adjustment, can be therapeutically equivalent in reducing LDL-C as concluded by Wlodarczyk et al. in a meta-analysis [15].

An equal reduction in TG levels was achieved with both atorvastatin and rosuvastatin. This is similar to results of the use of rosuvastatin versus atorvastatin in type 2 diabetes mellitus (URANUS) study comparing atorvastatin and rosuvastatin, both started at 10 mg daily, and the dose titrated up periodically till specific LDL-C goals were achieved [16]. Similarly, the superior benefit of aggressive lipid-lowering therapy for high-risk patients using statins (SUBARU) study and a study to verify the efficacy of rosuvastatin 5 mg as an aggressive lipid-lowering therapy for hypercholesterolemia (ASTRO-2) trial also failed to demonstrate a difference in ability of these two statins to lower the TG levels [8, 17]. On the contrary, rosuvastatin has been shown to produce greater reductions in TG levels as compared to atorvastatin in some other studies [13, 17].

A unique finding of this study is a reduction in HDL-C levels with both statins. Barakat et al. have earlier reported a similar phenomenon with rosuvastatin, atorvastatin, and pravastatin [18]. This is in contrast to the well-known fact that statins produce the modest elevations in HDL-C levels. Different statins vary in their HDL-C raising ability and the baseline HDL-C and TG levels are a predictor of statin-induced increases in HDL-C [19]. One possible explanation for these results is a high frequency of diabetics (75%) enrolled in this study, as diabetes is known to blunt the HDL-C response to statins. Poor compliance to treatment cannot be a reason since beneficial effects on LDL-C levels have been seen with both statins.

During the short study period, statins were tolerated very well by all the participants; none of them withdrew because of any disabling adverse event. Body aches and pains were observed but none of these patients had laboratory evidence of myositis. Liver function tests and serum creatine kinase levels were not routinely checked in all patients because of limited monetary resources and absence of sponsors for this study. The number of patients treated in this trial was very small relative to the already known estimated frequency of major side effects to statins. This, coupled with the fact that the patients were followed for six weeks only, means that the adverse effects profile of the two drugs is most likely to be different from what may be expected over a long term in day to day clinical practice.

The open-label study design might seem to be a short coming, but this problem was taken care of by the fact that randomization of every patient to a particular treatment group was done before the report of the baseline lipid levels was available. Moreover, the study relied on evaluation of objective rather than subjective evidence to compare the efficacy of the two drugs. Trials lasting for a longer duration are required to confirm whether the mathematical reduction in LDL-C levels seen in this study translates into a reduction in the number of atherosclerotic events in patients being treated with statins.

## 5. Conclusion

Rosuvastatin 5 mg has a greater effect on serum LDL-C reduction as compared to atorvastatin 10 mg. However, its effect on reduction of serum total cholesterol, TG, and HDL-C is the same as atorvastatin. Since there is no significant difference in side effect profile as well, rosuvastatin should be used in preference to atorvastatin.

## Figures and Tables

**Table 1:** Baseline characteristics of study population.

Variable	Atorvastatin group (*n* = 63)	Rosuvastatin group (*n* = 66)	*P* value
Age (years)	54.10 ± 12.66	55.44 ± 9.43	0.494
Gender (M : F)	34 : 29	26 : 40	0.097*
Education (years)	5.24 ± 3.63	3.70 ± 3.75	0.019
Current smokers	10 (15.87%)	3 (0.04%)	0.028*
BMI (kg/m^2^)	25.63 ± 3.66	27.61 ± 4.88	0.011
Waist circumference (cm)	94.49 ± 8.25	100.17 ± 9.87	0.001
Serum total cholesterol	4.92 ± 0.906	5.04 ± 0.91	0.447
Serum triglycerides	2.21 ± 1.10	1.99 ± 0.97	0.216
Serum HDL-C	0.9876 ± 0.20	1.20 ± 0.93	0.071
Serum LDL-C	2.91 ± 0.86	3.19 ± 0.92	0.074

*By chi-square test; all other *P* values were calculated by independent samples *t*-test.

**Table 2:** Underlying diagnosis.

Disease	Atorvastatin group (*n* = 63)	Rosuvastatin group (*n* = 66)
Diabetes mellitus	53	44
Essential hypertension	27	24
Effort angina	4	7
Congestive heart failure	2	4
MI in past	1	3
Stroke in past	2	3

Figures refer to the number of patients. Many patients had more than one diagnosis.

**Table 3:** Mean reduction in levels of different lipid fractions.

Variable	Atorvastatin group (*n* = 63)	Rosuvastatin group (*n* = 66)	*P* value*
Absolute reduction from baseline (mmol/L)			
Serum total cholesterol	0.77 ± 0.85	1.09 ± 1.10	0.065
Serum triglycerides	0.31 ± 1.01	0.24 ± 0.92	0.657
Serum HDL-C	0.10 ± 0.25	0.29 ± 0.94	0.131
Serum LDL-C	0.54 ± 0.88	0.96 ± 0.96	0.011

Percent reduction from baseline (%)			
Serum total cholesterol	13.82 ± 17.10	19.84 ± 19.21	0.062
Serum triglycerides	5.92 ± 40.48	3.52 ± 37.26	0.727
Serum HDL-C	7.63 ± 24.47	5.76 ± 38.14	0.742
Serum LDL-C	13.66 ± 27.99	24.34 ± 31.79	0.045

*P values refer to the comparison between treatment groups.
